# The Responsibility of Names

**Published:** 1915-06-26

**Authors:** 


### THE RESPONSIBILITY OF NAMES.

Names and phrases are perhaps hardly affairs of the first order, and just now it must be admitted that the atmosphere of each of us is filled with a tremendous sense of facts. Yet names stand for a good deal, and they are very apt to become our masters, more especially when time has given them a firm root. Again, there is something due on the score of dignity. Language has its æsthetic as well as its utilitarian claim, and loose phrasing is very apt to indicate loose thinking, and even to breed it. Therefore, much may be said for the well-adjusted and right use of words. It promotes clearness of thought, accuracy of description, and effectiveness in speech. At the same time, it recognises the debt we owe to our mother tongue and to those who are to succeed us in its heritage. These latter merit our protection from the tyranny of careless and misleading phrases.

As a first example of inaccurate terminology we will take the term "iodide of potash." It is often heard on medical tongues, and is frequently written by authors who ought to know better. Yet, without any question, it is quite incorrect. The salt which is indicated by this term is formed by the union of a metal, potassium, with the halogen element iodine, and the correct name of the compound is, therefore, potassium iodide or iodide of potassium. There is no question at all of "potash," which is not potassium, but the oxide of potassium. Hence the use of the term "iodide of potash" is an announcement either of ignorance or of carelessness. There may be some excuse for such phrases as "carbonate of potash," "citrate of potash," and so on, because a possible view of the chemical structure of these salts is that they are produced by the union of "potash" with the corresponding acids. Such a view once held the field, though it is now out of date. Hence the worst that can be said against these terms is that they are old-fashioned and behind the times. But "iodide of potash" suffers a much more serious indictment. It is not only incorrect to-day, but it always has been incorrect. The suggestion of the phrase is hopelessly vicious, and altogether inexcusable. It deserves the pillory, and this is where we wish to place it. Such other phrases as we have mentioned may, perhaps, be passed by with the forbearance due to those whose light is that of a departed day. But "iodide of potash" is a terminological outrage, undeserving of leniency and wholly beyond pardon. Some will say, "What does it matter?" Such a question is surely an aggravation of the offence. To ask whether it matters to be accurate indicates the corrupting influence of careless thinking. Those who propose such a question are, it is to be feared, beyond the reach of the secular arm, and must be left to the influence of more charitable intercessions.

Another point for criticism in the terms sometimes heard in medical circles is the habit of blunt and inelegant contractions. There is something even more heartrending than "iodide of potash." It is "pot. iod." In the same criminal group are such phrases as "dip." and "syph.," and the like—phrases which chill the blood and shatter the nerves of anyone who cherishes some sense of the dignity and worth of language. The pernicious habit readily spreads to the young generation, as is readily seen by anyone who suffers the discipline of students' reports. Indeed, the relatively moderate offence of the master seems to act as a stimulus to the perverted ingenuity of the pupil, who, under such provocation, produces contractions of truly horrifying proportions. The process seems inexplicable except on the assumption of the truth of the doctrine of original sin. Here, then, is a firm plea for care in the use of words. Neglect means the fruitful influence of a bad example, and the seed multiplies into a large and varied crop. The tares are bad in themselves; and they have this further disaster—they choke the wheat. Possibly when both are already in existence it may be necessary to leave them "until the harvest," and each to its appropriate fate. But the best plan of all, obviously, would be to keep out the tares from the very beginning.

In still another respect we suggest that our medical writings might be improved—namely, in holding steadily to the distinction between facts and inferences. What is actually seen, heard, felt, and noted by a competent observer is always a welcome record, but conclusions presented without the facts on which they rest have little more than personal value, and, in any event, they ought not to be offered as statements of fact. Even as we write there comes a hospital report stating that "the house physician noted that optic atrophy was present," the true position, we presume, being that the house physician noted certain facts, and on these, whether justly or unjustly we have no means of knowing, rested his opinion of the existence of atrophic changes in the optic discs. To present the house physician's opinion about the facts as a substitute for the facts themselves is a blunder in form as well as in substance. What an individual "thinks" may be of surpassing interest to his family circle, but a less partial audience is concerned with the reasons—if any—on which the "thinking" is based. Medical practitioners, with their special training in the importance of accuracy in statement, have a great opportunity to set a good example in the careful use of language. May we gently hint that this opportunity is not always fully utilised?

